# A highly conserved sequence of the viral TAP inhibitor ICP47 is required for freezing of the peptide transport cycle

**DOI:** 10.1038/s41598-017-02994-5

**Published:** 2017-06-07

**Authors:** Tony Matschulla, Richard Berry, Carolin Gerke, Marius Döring, Julia Busch, Jennifer Paijo, Ulrich Kalinke, Frank Momburg, Hartmut Hengel, Anne Halenius

**Affiliations:** 1 Institute of Virology, Medical Center – University of Freiburg, Freiburg, Germany; 2 Faculty of Medicine, University of Freiburg, Hermann-Herder-Str 11, 79104 Freiburg, Germany; 3 Infection and Immunity Program, Dept. of Biochemistry and Molecular Biology, Biomedicine Discovery Institute, Monash University, Clayton, Victoria Australia; 4 ARC Centre of Excellence in Advanced Molecular Imaging, Monash University, Clayton, Victoria Australia; 5 Spemann Graduate School of Biology and Medicine (SGBM), Albert-Ludwigs-University, Freiburg, Germany; 6 Institute for Experimental Infection Research, TWINCORE, Centre for Experimental and Clinical Infection Research, a joint venture between the Helmholtz Centre for Infection Research and the Hannover Medical School, Feodor-Lynen-Str. 7, 30625 Hannover, Germany; 7 Institute of Virology, University of Duesseldorf, Universitaetsstr. 1, 40225 Duesseldorf, Germany; 8 Clinical Cooperation Unit, Applied Tumor Immunity, Antigen Presentation and T/NK Cell Activation Group, German Cancer Research Center, Im Neuenheimer Feld 280, 69120 Heidelberg, Germany

## Abstract

The transporter associated with antigen processing (TAP) translocates antigenic peptides into the endoplasmic reticulum (ER) lumen for loading onto MHC class I molecules. This is a key step in the control of viral infections through CD8+ T-cells. The herpes simplex virus type-1 encodes an 88 amino acid long species-specific TAP inhibitor, ICP47, that functions as a high affinity competitor for the peptide binding site on TAP. It has previously been suggested that the inhibitory function of ICP47 resides within the N-terminal region (residues 1–35). Here we show that mutation of the highly conserved _50_PLL_52_ motif within the central region of ICP47 attenuates its inhibitory capacity. Taking advantage of the human cytomegalovirus-encoded TAP inhibitor US6 as a luminal sensor for conformational changes of TAP, we demonstrated that the _50_PLL_52_ motif is essential for freezing of the TAP conformation. Moreover, hierarchical functional interaction sites on TAP dependent on _50_PLL_52_ could be defined using a comprehensive set of human-rat TAP chimeras. This data broadens our understanding of the molecular mechanism underpinning TAP inhibition by ICP47, to include the _50_PLL_52_ sequence as a stabilizer that tethers the TAP-ICP47 complex in an inward-facing conformation.

## Introduction

To enable the immune system to recognize infected cells, the intracellular protein content is sampled as peptides on the cell surface by MHC class I molecules. Whereas self-peptides are typically ignored, antigenic peptides are recognized by surveilling cytotoxic CD8+ T-lymphocytes, which subsequently become activated and eliminate the infected cell. The MHC class I peptide ligands are produced in the cytosol by proteasomal degradation. Therefore, for loading onto MHC class I molecules the peptides have to cross the endoplasmic reticulum (ER) membrane by active transport. This process is performed by the transporter associated with antigen processing (TAP)^1^.

TAP belongs to the large family of ATP binding cassette (ABC) transporters that are responsible for translocation of a broad spectrum of substrates across cellular membranes. The characteristic structure of ABC transporters comprises two transmembrane domains (TMD) and two nucleotide binding domains (NBD). The TAP complex is heterodimeric, being composed of TAP1 and TAP2 subunits, each of which comprises a NBD and a TMD. The TMD spans the ER membrane 10 times^2, 3^, with the six C-terminal transmembrane helices (TM1-6) of each subunit together forming the core of the peptide translocation pore^4^. The N-terminal TMs, or TMD0, link TAP to the MHC class I peptide loading complex by binding to the chaperone tapasin^4–7^. The TMD0 is dispensable for peptide transport^4, 5^, but the interaction with tapasin is crucial for efficient MHC class I loading^8^.

The NBDs bind and hydrolyze ATP, which is absolutely required to accomplish active and unidirectional peptide transport^9–11^, a process involving precisely coordinated major structural rearrangements of the TMDs and the NBDs^10^. A transmission interface between the NBDs and TMDs is formed by the so-called coupling helices (CH1 and CH2), which are located in the extended cytosolic loops between the TMs 2 and 3 and TMs 4 and 5^12^. However, TAP1 and TAP2 are heterologous, possessing approximately 33% sequence homology and play different roles during the translocation cycle^13, 14^. TAP1 contains a degenerate ATP binding site with non-canonical variations in the Walker A and B motifs, rendering ATP binding and hydrolysis by the Walker A and B motifs of TAP2 crucial for the peptide translocation cycle^15–17^.

Large DNA viruses have developed numerous strategies to interrupt the MHC class I antigen presentation pathway. To date five virally encoded inhibitors have been reported to directly block TAP function: ICP47 (herpes simplex virus type-1, HSV-1)^18, 19^, US6 (human cytomegalovirus, HCMV)^20–22^, BNLF2a (Epstein-Barr virus, EBV)^23^, UL49.5 (varicelloviruses)^24^, and CPXV12 (poxvirus)^25^. Initially observed as an HSV-1 early gene product inhibiting antigen presentation to CD8^+^ T-cells^26^, ICP47 was the first TAP inhibitor described^18, 19^. ICP47 is a small (88 amino acids (aa)) protein that is localized within the cytosol^26^, where it is considered to function as a high affinity competitor^27, 28^ that blocks peptide binding to TAP. Notably, ICP47 is highly species specific, and can effectively inhibit human but not mouse TAP^19, 27, 28^. Functional analysis of ICP47 truncation mutants revealed that the amino acids 2–35 are sufficient for efficient inhibition of peptide translocation^29, 30^. The structure of ICP47-bound TAP has been resolved by cryo-EM analysis to an overall resolution of 6.5 Å^31^. TAP adopted a structure very similar to other ABC transporters such as MsbA and P-glycoprotein and was locked in an inward-facing conformation due to its interaction with ICP47. More specifically, the region of ICP47, that was modeled in to the electron density map (residues 3–50), adopted a helix-turn-helix hairpin-like structure that wedged into the closed pore formed by the core TMs 1–6 of TAP1 and TAP2. Here, the high binding affinity of ICP47, which exceeds that of peptide substrates^27^ was attributed to the buried surface of the TAP-ICP47 interface. However, precisely how ICP47 so efficiently holds TAP in an inward-facing conformation remains unclear.

Although the N-terminal segment of ICP47 is sufficient to block peptide binding to TAP, it has been previously suggested that sequences located at the central or in the C-terminal segment of ICP47 could also be important for TAP inhibition^29^. Indeed, the most conserved sequence of ICP47 and its homologs within the alpha-herpesvirus family is found in the central portion of the protein (aa 34–52)^32^. We now present data demonstrating that this region of ICP47 is responsible for freezing of the TAP conformation. We were able to narrow down the responsible residues of ICP47 to the positions 50–52 (ProLeuLeu), which are localized in close proximity to the TAP2 CH1 coupling helix. Here we propose a revision of the mechanism of ICP47-mediated TAP inhibition, in which the N-terminus (1–35) prevents peptide binding to TAP, but the _50_PLL_52_ motif can induce freezing of TAP conformation and thereby block its function completely.

## Results

### The highly conserved central region of ICP47 is essential for its inhibitory function

**Figure 1:**
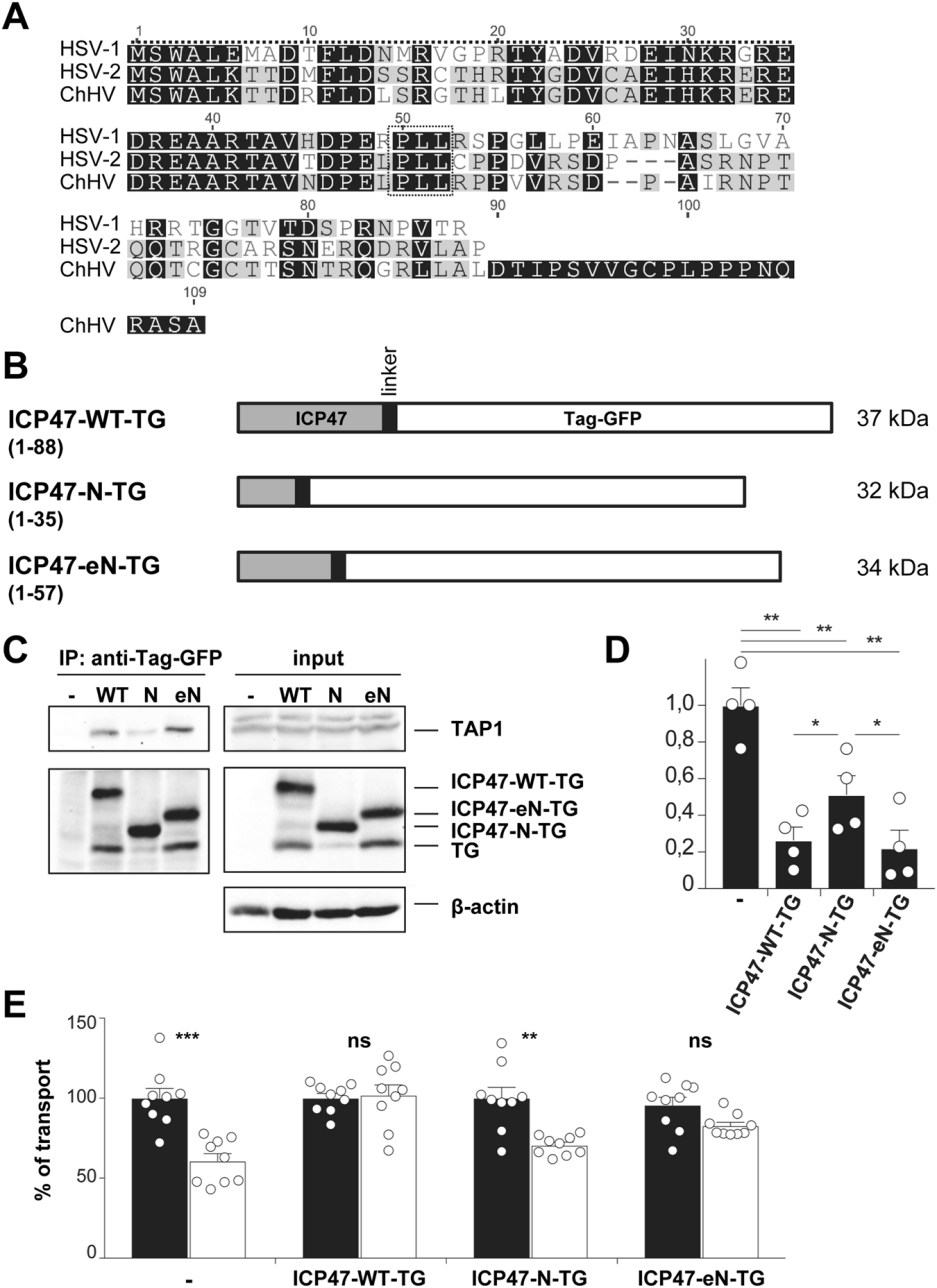
The ICP47 residues 1–35 are not sufficient for efficient inhibition of TAP. (**A**) Sequence alignment of ICP47 sequences from HSV-1, HSV-2 and chimpanzee alpha-1 herpesvirus (ChHV). Identical amino acids are shown in black. The dotted lines indicate the residues 1–35, that were shown to be sufficient for TAP inhibition^29, 30^. The highly conserved residues 50–52 are marked with a box. (**B**) Schematic depiction of ICP47-TagGFP fusion proteins with number of N-terminal ICP47 residues in front of the linker and TagGFP, and apparent protein sizes. (**C**) HeLa cells stably expressing fusion proteins shown in B were lysed in digitonin lysis buffer and an anti-TagGFP IP was performed. Aliquots of the lysates (input) and recovered proteins (IP) were separated by SDS-PAGE and detected by Western blotting (TAP1, 148.3; ICP47-TG fusion constructs, anti-TagGFP). One representative experiment out of three independent experiments is shown. Shown blots are cropped. Original blots are depicted in Fig. S3. The same procedure for presentation of blots has been used in all following figures. (**D**) Cells from C were analyzed by FACS using W6/32 mAbs for surface expression analysis. The diagram shows relative MFI values compared to control cells (−). The error bars indicate the standard error of mean, the circles indicate individual samples derived from independent experiments (unpaired student *t*-test, *p ≤ 0.05, **p ≤ 0,001 n = 4). (**E**) HeLa cells stably transduced with ICP47-TG constructs were semi-permeabilized with streptolysin O (SLO) and a peptide translocation assay was performed with a fluorescent peptide (5 nM of NST-Cy5) and either ATP (black bars) or ADP (white bars) (10 mM each). Cells were analyzed by flow cytometry for Cy5 mean fluorescence intensity (MFI). The mean MFI value of untransduced Hela cells with ATP was set as 100% and used to calculate relative translocation for all samples (shown as % of transport). The error bars indicate the standard error of mean, the circles indicate individual samples of 3 independent experiments (Kruskal-Wallis test with Dunn’s correction for multiple comparison, **p ≤ 0.007, ***p ≤ 0,0008 n = 9).

Almost 20 years ago two independent studies suggested that the residues 2–35 of ICP47 are just as efficient ﻿in inhibiting﻿ TAP as the full-length protein^29, 30^. In these studies ICP47 was produced synthetically or was recombinantly expressed and analyzed using microsomes or permeabilized cell systems. These findings have never subsequently been confirmed in intact cells. We hypothesized that the highly conserved region of ICP47 (aa 34–52, Fig. 1A) might be important for the stability of interaction of ICP47 with TAP in a cellular context. To investigate this we first attempted to express short ICP47 truncation mutants. However, we failed to express ICP47 aa 1–35 with a C-terminal HA-tag despite applying several different expression systems, and we never observed any effect on MHC class I surface expression (data not shown). Although it is possible to synthesize functional ICP47 1–35 peptides, apparently this mini-protein was not stable with a C-terminal HA-tag. Therefore, we generated fusion proteins with a linker sequence and TagGFP attached to three variants of ICP47: the wild-type ICP47 (WT-TG; aa 1–88), the N-terminus (N-TG; aa 1–35) and the extended N-terminus (eN-TG; 1–57) (Fig. 1B). These constructs were stably transduced into HeLa cells and protein expression was analyzed by Western blotting (Fig. 1C, right panel). All fusion proteins were expressed to similar levels, while some expression of isolated TagGFP was also observed which was probably due to internal transcriptional insertion. To investigate whether the fusion proteins were able to bind to TAP co-immunoprecipitation (co-IP) experiments using an anti-TagGFP antibody were performed. The fusion of TagGFP to ICP47 did not impair TAP binding since the WT-TG construct was able to co-immunoprecipitate TAP1 (Fig. 1C, left panel). However, despite stable expression the N-TG construct repeatedly showed a reduced level of TAP binding, while the eN-TG and ICP47 WT-TG constructs bound TAP with similar strengths. Overall, this data suggested that the ICP47 residues 36–57 are important for stable TAP binding.

To analyze whether the strength of TAP co-immunoprecipitation was mirrored by the degree of TAP inhibition, we analyzed MHC class I expression on the cell surface as a surrogate marker for TAP function. As suggested by the co-IP, the WT- and eN-TG constructs strongly downregulated MHC class I, whereas the N-TG fusion protein was markedly less effective in blocking MHC class I expression (Fig. 1D). This difference was even more prominent when measuring surface expression of a transiently transfected MHC class I molecule (Figs S1 and S2B). TagGFP itself had no effect on MHC class I expression or TAP binding as demonstrated using an N-terminally truncated non-functional ICP47 fusion protein (Fig. S2). In agreement with the level of MHC class I expression, ICP47-WT-TG abrogated TAP dependent peptide transport in HeLa cells, whereas ICP47-N-TG did not. ICP47-eN-TG expressing HeLa cells showed merely residual TAP-dependent peptide translocation, which was reduced compared to ICP47-N-TG expressing cells (Fig. 1E). In conclusion, the highly conserved region in the central part of ICP47 has profound effect on ICP47’s inhibitory capacity.

### The central region of ICP47 is required to block conformational changes of TAP

**Figure 2:**
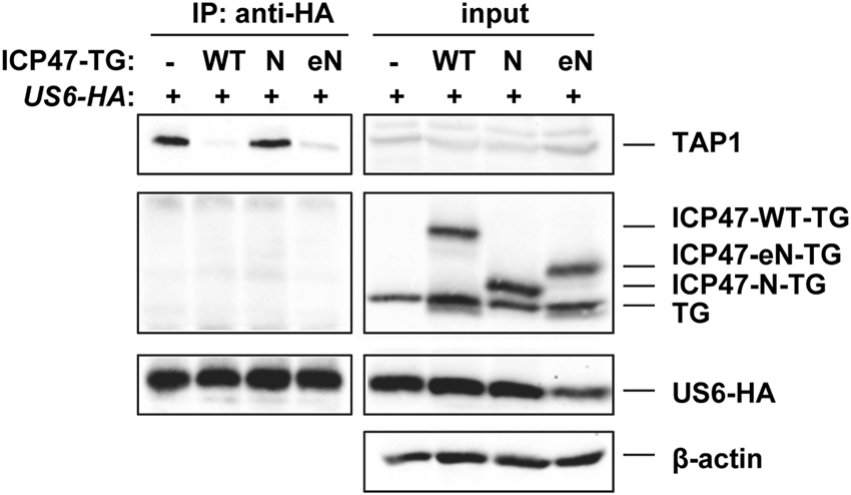
The central part of ICP47 is required to block conformational changes of TAP. HeLa cells stably expressing fusion proteins as indicated were transiently transfected with a US6-HA expression plasmid. At 20 h post-transfection cells were lysed in digitonin lysis buffer and an IP was performed using anti-HA antibodies. Aliquots of the lysates (input) and recovered proteins (IP) were separated by SDS-PAGE and detected in Western blot (TAP1, 148.3; ICP47-TG fusion constructs, anti-TagGFP; US6-HA, anti-HA). One representative experiment out of three independent experiments is shown.

ICP47 interacts with TAP in a conformation that is receptive to peptide binding from the cytosolic side of the ER membrane^31, 33^, termed the inward-facing conformation. In contrast to ICP47, it has been proposed that the HCMV encoded luminal TAP inhibitor US6 binds to the outward-facing conformation of TAP, after release of peptide substrates on the luminal side^3, 33^. This suggests that US6 could be used as a sensor for conformational changes during the TAP cycle. Therefore, we transiently transfected C-terminally HA-tagged US6 into HeLa control cells or cells stably expressing ICP47 fusion proteins followed by co-IP using anti-HA antibodies. In control cells, tagged US6 remains Endoglycosidase H sensitive, binds to TAP and down-regulates MHC class I cell surface expression (Fig. 2, left panel and Fig. S4). However, in the presence of ICP47-WT-TG fusion protein, US6 was not able to bind to TAP. The structural model of ICP47 and TAP suggests that access to the TAP pore via the ER luminal side is restricted^34^ and therefore it is most unlikely that US6 binding to TAP is blocked due to overlapping interaction sites with ICP47. In all likelihood, stable binding and freezing of TAP in an inward-facing conformation by ICP47 resulted in occlusion of the US6-binding site on the luminal side.

A similar effect on US6 binding was also observed for the eN-TG construct. In contrast, the N-terminal fusion construct did not block US6 binding to TAP. Overall, these data suggests that the central region of ICP47 (residues 36–57) somehow retains TAP in an inward-facing state.

### The highly conserved sequence _50_PLL_52_ is required for freezing of the TAP conformation

**Figure 3:**
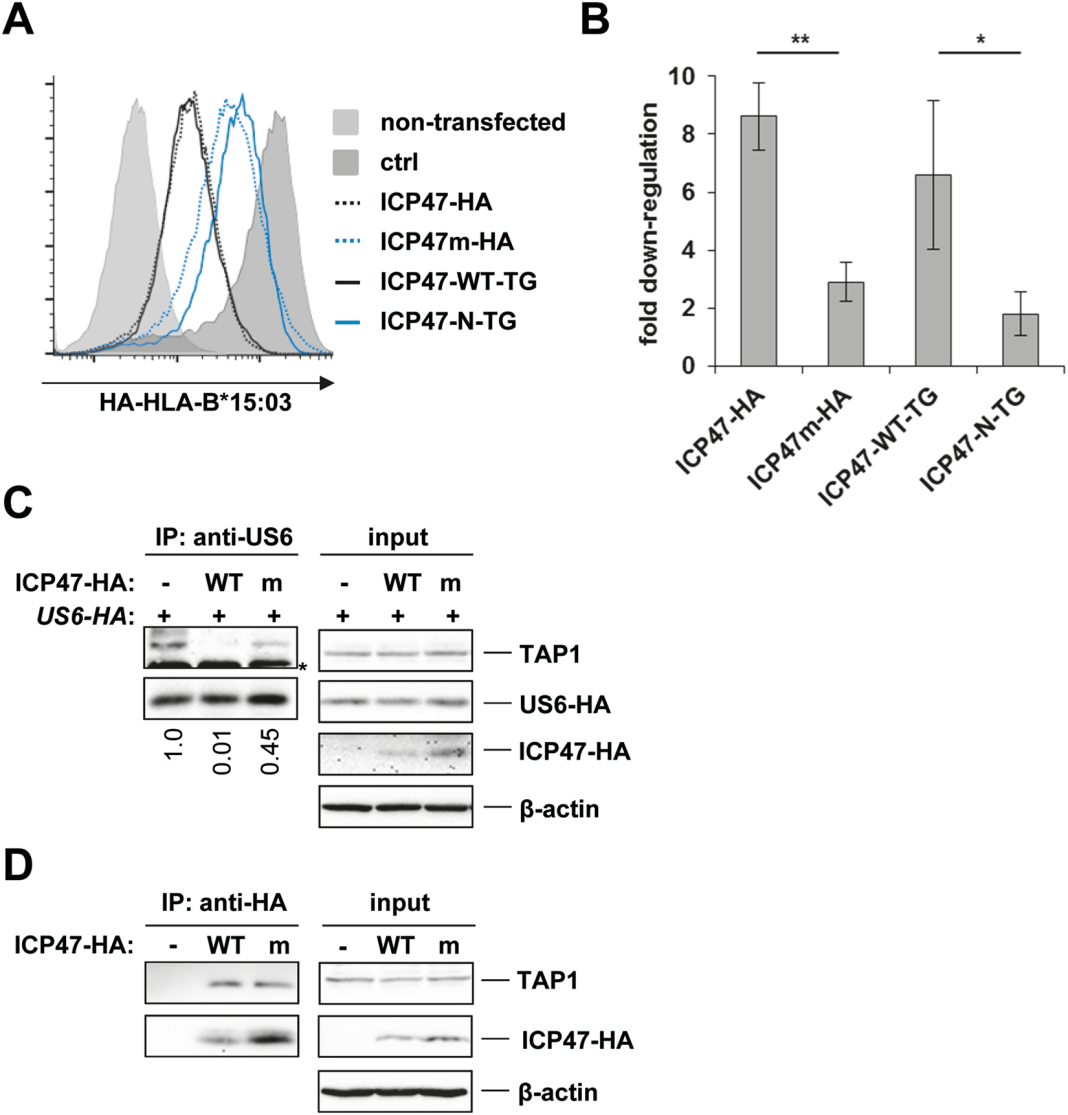
The highly conserved ICP47 residues 50–52 are required for freezing of the TAP conformation. (**A**) HeLa cells were transiently transfected with HA-HLA-B*15:03, a control (ctrl) or ICP47 constructs as indicated and a plasmid encoding EGFP for gating of transfected cells. At 20 h post-transfection HA-HLA-B*15:03 surface expression was analyzed by FACS using anti-HA antibodies. One representative experiment out of four independent experiments is shown. (**B**) Relative downregulation of HA-HLA-B*15:03 was determined by dividing the MFI value of the control sample by the MFI of indicated samples. Mean values of four independent experiments are shown. Statistical analyses were performed applying one-way analysis of variance, ANOVA (Bonferroni), *P < 0.05. (**C**) Control HeLa cells (−) or HeLa cells stably expressing WT or mutant (m) ICP47-HA were transiently transfected with US6-HA. At 20 h post-transfection cells were lysed in digitonin lysis buffer and an IP was performed an anti-US6 antiserum. Recovered proteins (IP) and aliquots of the lysates (input) were separated by SDS-PAGE and detected in Western blot (TAP1, 148.3; ICP47-HA and US6-HA, anti-HA). The relative intensity of the TAP1 band compared to the US6-HA band in the IP samples is given below the upper panel. The value of the control sample was set to 1. One representative experiment out of three independent experiments is shown. The asterisk indicates the HC of the antibody used for IP. (**D**) HeLa cells stably expressing ICP47-HA or ICP47m-HA were lysed in digitonin lysis buffer and an IP was performed using an anti-HA antibody. Detection was performed as in C. One representative experiment out of two independent experiments is shown.

Our findings suggest that the N-terminus of ICP47 (1–35) expressed as a fusion protein is able to bind to TAP, but this binding is not sufficient to efficiently block TAP function in a cellular context. Instead, complete arrest of the transport activity was only observed when the amino acids 36–57 were also present. To exclude the possibility that the loss of function of ICP47-N-TG was due to conformational changes induced by the C-terminal linker sequence, we performed site-directed mutagenesis on ICP47 fused to a C-terminal HA-tag (ICP47-HA). In a previous study, in which the affinity of various synthetic C-terminally truncated ICP47 fragments to TAP was determined, a noticeable difference in affinity was observed between the fragments comprising the aa 1–48 and 1–53 with K_d_ values of 126 and 50 nM, respectively^30^. This suggested that the residues 49–53 were able to increase the affinity of ICP47 for TAP. Notably, this region contained three highly conserved residues (Pro_50_Leu_51_Leu_52_, Fig. 1A), which we mutated to AlaAlaAla (ICP47m-HA). Stable expression of both WT ICP47-HA and ICP47m-HA was detected after transient transfection of HeLa cells (Fig. S5A). To evaluate the impact of the residues 50–52 on TAP inhibition, cell surface expression of a co-transfected HA-tagged MHC class I molecule (HA-HLA-B*15:03) was assessed in HeLa cells that were transiently transfected with ICP47-HA, ICP47m-HA, ICP47-WT-TG and ICP47-N-TG. Note that in this experiment, only HA-tagged MHC class I molecules, not HA-tagged ICP47 was detectable at the cell surface, as evidenced by the absence of staining in samples including untagged HLA-B*44:02 (Fig. S5B). Whereas the ICP47 WT constructs (ICP47-HA and ICP47-WT-TG) strongly downregulated HA-HLA-B*15:03, the mutants were similarly impaired in their ability to block HA-HLA-B*15:03 expression (Fig. 3A,B). This suggested that the ICP47m-HA mutant functionally mimicked the ICP47-N-TG fusion protein.

To test whether residues 50–52 are required for a full arrest of conformational cycling of TAP, HeLa cells were stably transduced with the ICP47-HA constructs and then transiently transfected with US6-HA. Co-IP experiments were then performed using anti-US6 antiserum, and TAP binding to US6 was visualized by detection of TAP1 via Western blot. As expected, WT ICP47-HA blocked US6 binding to TAP (Fig. 3C), however, the ICP47m-HA mutant was reproducibly less efficient in preventing US6 binding to TAP (see quantification of TAP1 co-IP in Figs 3C and S5C). As observed after transient transfection (Fig. S5A), ICP47m-HA was more stably expressed than WT ICP47-HA (Figs 3C,D and S5D). ICP47m-HA was also clearly able to bind to TAP as shown by co-IP of TAP1 (Fig. 3D). In conclusion, these data confirm that the conserved residues _50_PLL_52_ in the central part of ICP47 are involved in the freezing of the TAP conformation.

### Interaction with the human TAP2 subunit is absolutely required for ICP47 mediated inhibition

Next we sought to investigate whether residues 50–52 of ICP47 influence the manner by which ICP47 interacts with TAP^19, 27^. Taking advantage of the well-documented species specificity of ICP47^15, 23^, we set out to analyze the influence of WT ICP47-HA and ICP47m-HA on mixed combinations of human (h) and rat (r) TAP subunits (hT1, hT2, rT1, rT2). We used a rat TAP1 subunit with a human C-terminus (starting in the NBD at residue 539) to allow for detection by the anti-human TAP1 mAb 148.3. The TAP2 subunits were tagged with a FLAG-epitope at the C-terminus. TAP1 and TAP2 subunits were expressed in TAP-deficient mouse CMT64.5 cells by lentiviral transduction using the selection markers puromycin and neomycin, respectively. Subsequently ICP47-HA and ICP47m-HA were transduced using a bicistronic construct encoding *EGFP* to allow gating on ICP47 expressing cells during FACS analysis.

**Figure 4:**
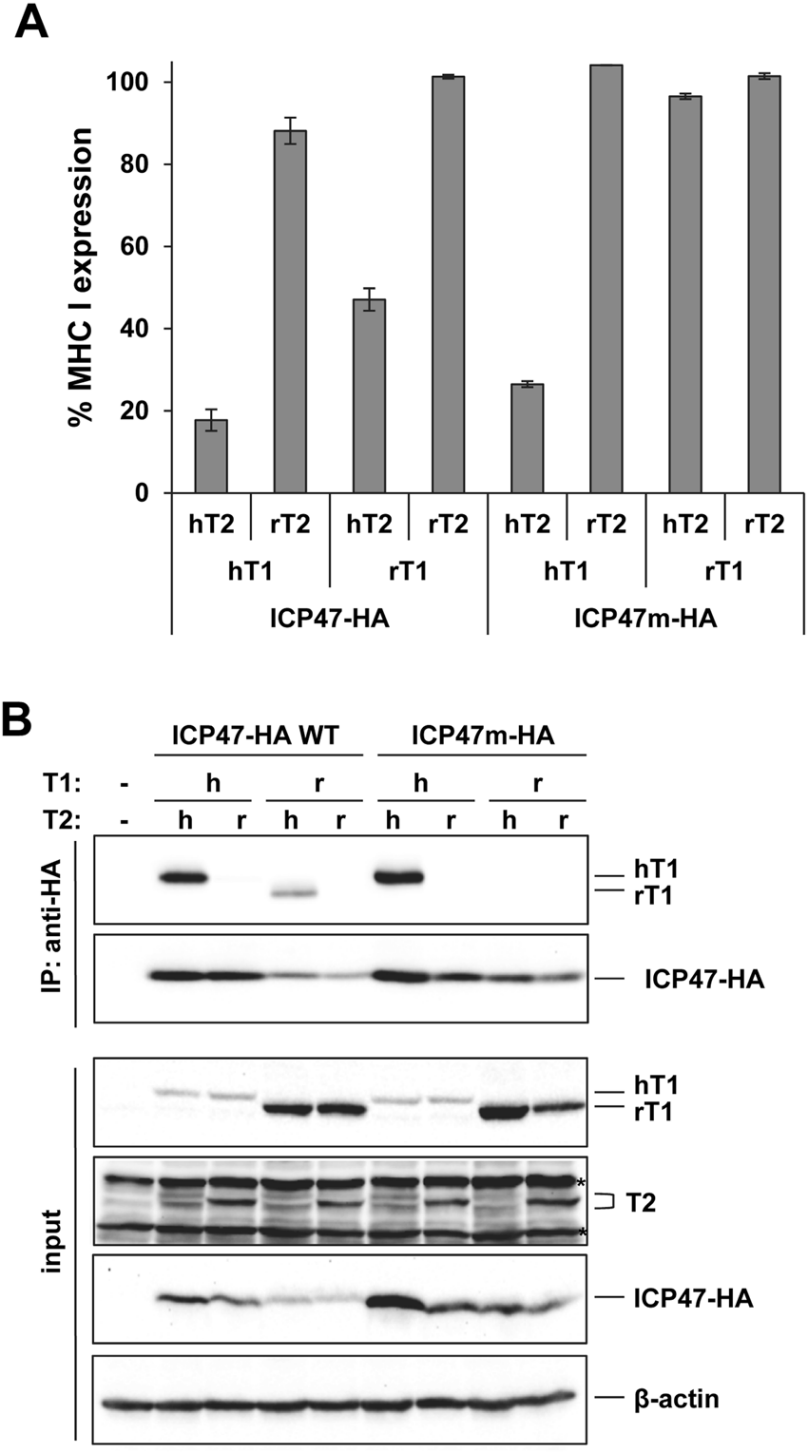
Interaction with the human TAP2 subunit is required for inhibition by ICP47. (**A**) Mouse CMT64.5 cells stably expressing TAP1, TAP2 and ICP47-HA or ICP47m-HA were analyzed by flow cytometry using the MHC class I specific antibody 20-8-4 recognizing H2-K^b^ and H2-D^b^. The diagram shows relative MFI values of EGFP positive cells (ICP47 positive) compared to the EGFP negative (ICP47 negative) cells in the same sample. Mean values of two independent experiments is shown. (**B**) The cells from A were lysed in digitonin lysis buffer and an IP was performed using anti-HA antibodies. Aliquots of the lysates (input) and recovered proteins (IP) were separated by SDS-PAGE and detected in Western blot (TAP1, 148.3; ICP47-HA, anti-HA; TAP2, anti-FLAG). Original blots are shown in Fig. S7B. A replicate of the experiment is shown in Fig. S8.

Different combinations of human and rat TAP1 and TAP2 subunits induced the expression of MHC class I to a similar extent on the CMT64.5 cells (Fig. S6A,B). Expression of ICP47-HA in these cells confirmed the species-specific inhibition by blocking the function of human TAP subunits (hT1/hT2, 17% expression with ICP47 compared to without) but not of rat subunits (rT1/rT2, 101% expression, Fig. 4A). Interestingly, when combining TAP subunits from the two species only the combination of rT1/hT2 was appreciably regulated by ICP47 (intermediate inhibition, 47% expression), whereas hT1/rT2 expression resulted in only limited MHC class I reduction (88% expression). In conclusion, whereas human TAP2 was more critical for TAP inhibition than TAP1, interaction with human TAP1 was still required for full inhibition by ICP47.

The inhibition of hT1/hT2 by ICP47m-HA was surprisingly strong in this system (Fig. 4A, 26% expression) compared to the loss of function observed in HeLa cells (Fig. 3A,B). A possible explanation for this could be a suboptimal compatibility between human TAP molecules and chaperons in the mouse CMT64.5 cells, which could affect the regulation by WT and mutated ICP47. The TAP sequences themselves might also differ between the two systems. Whereas the human TAP subunits TAP1A and TAP2E^3^ were overexpressed in the CMT64.5 cells, the TAP sequences in HeLa cells are not known. This issue should be revealed in future experimental efforts.

While the ICP47m-HA mutant showed a species-specific regulation of TAP, no inhibition of mixed TAP subunits (including rT1/hT2) was observed. This indicated that without the _50_PLL_52_ sequence, impairment of peptide transport via human TAP2 was not possible, emphasizing the importance of this sequence for the inhibitory function.

Next, we analyzed binding of ICP47-HA to combinations of human and rat TAP subunits by co-IP and found a clear correlation between the extent of inhibition and binding. Transduction levels of the ICP47-HA constructs varied, as indicated by the relative intensity of the bands detected by Western blot analysis (Fig. 4B). Combination of human TAP subunits resulted in clear detection of TAP1 in Western blot after immunoprecipitating with an anti-HA antibody (Fig. 4B). Consistent with the functional analysis, an interaction with rT1/hT2 was observed for ICP47-HA, but not for ICP47m-HA. Long exposure of the Western blot membrane did not change this result (Fig. S7A). Of note, the rat TAP subunits were expressed at higher levels in the CMT64.5 cells than the human TAP subunits (Fig 4B, input), perhaps because rat TAP subunits are better stabilized by chaperons in these mouse cells. However, due to the fact that TAP complexes were not co-immunoprecipitated in the combinations hT1/rT2 and rT1/rT2, we excluded the possibility that the low inhibition rate of rat TAP subunits could be due the strong overexpression.

### Hierarchical impact of ICP47 interactions sites on human TAP1 and TAP2

**Figure 5:**
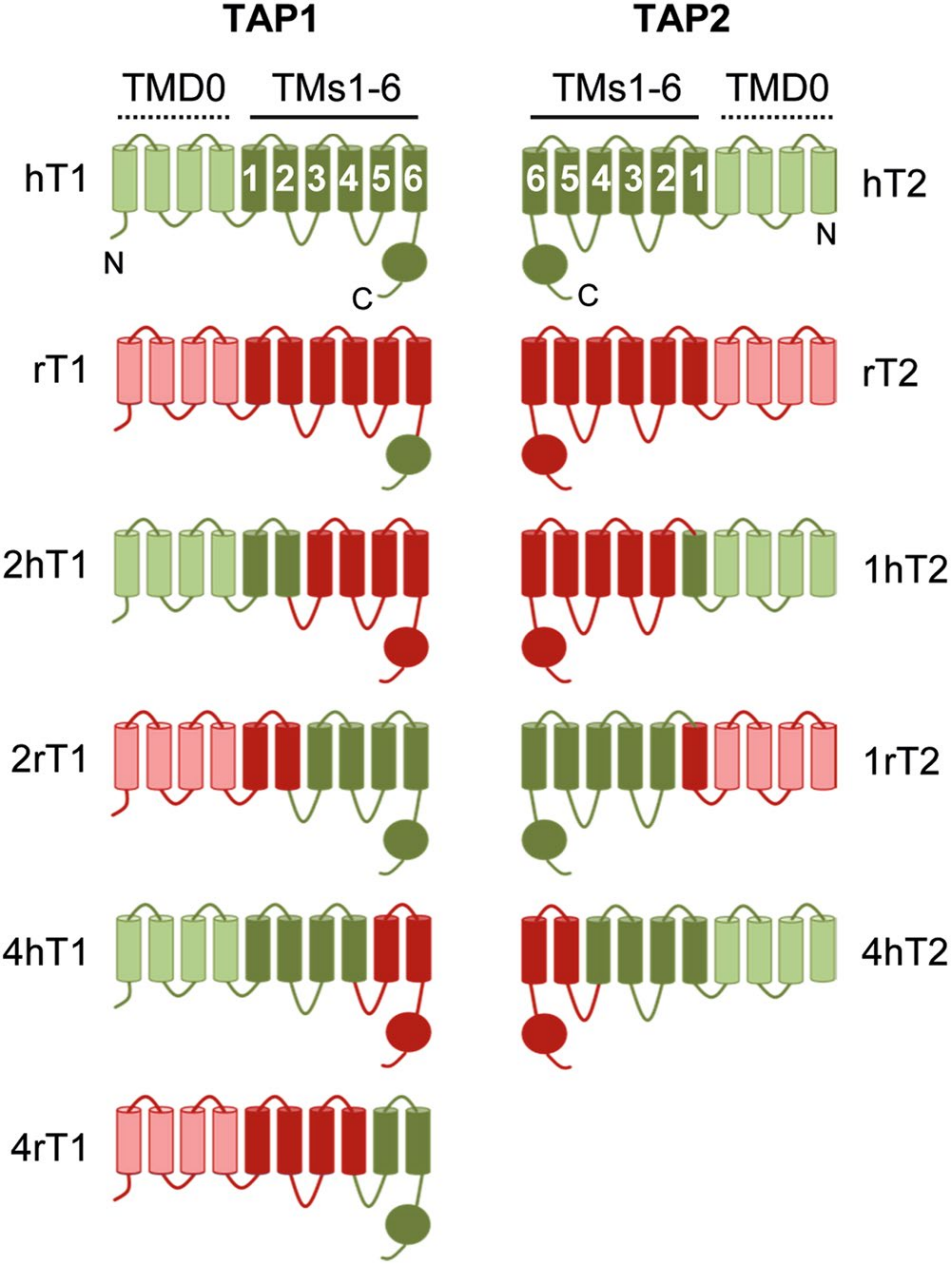
A schematic depiction of TAP1 and TAP2 human/rat chimeras. Human sequences are shown in green and rat sequences in red. TMs of the TMD0 are shown in a lighter color.

To gain a deeper insight into which interactions are important for mediating inhibition of TAP by ICP47, we analyzed human/rat chimeric subunits of TAP1 and TAP2. We have previously utilized a similar approach with a US6 mutant (Cys127Tyr) that renders this inhibitor human TAP-specific and thus identified several US6 interaction sites on TAP1 and TAP2^3, 35^. We used the same chimeric constructs as in our previous study^3^, but now renamed them (Supplementary Table 1) to allow for a convenient comparison with the published structural model of ICP47 and TAP^30^. The names of the chimeras indicate the number of the N-terminal core TMs of a specific species, e.g. 1rT2 is a TAP2 molecule with a rat N-terminus including TM1, whereas TMs 2–6 and the C-terminus are derived from human TAP2 (Fig. 5).

**Figure 6:**
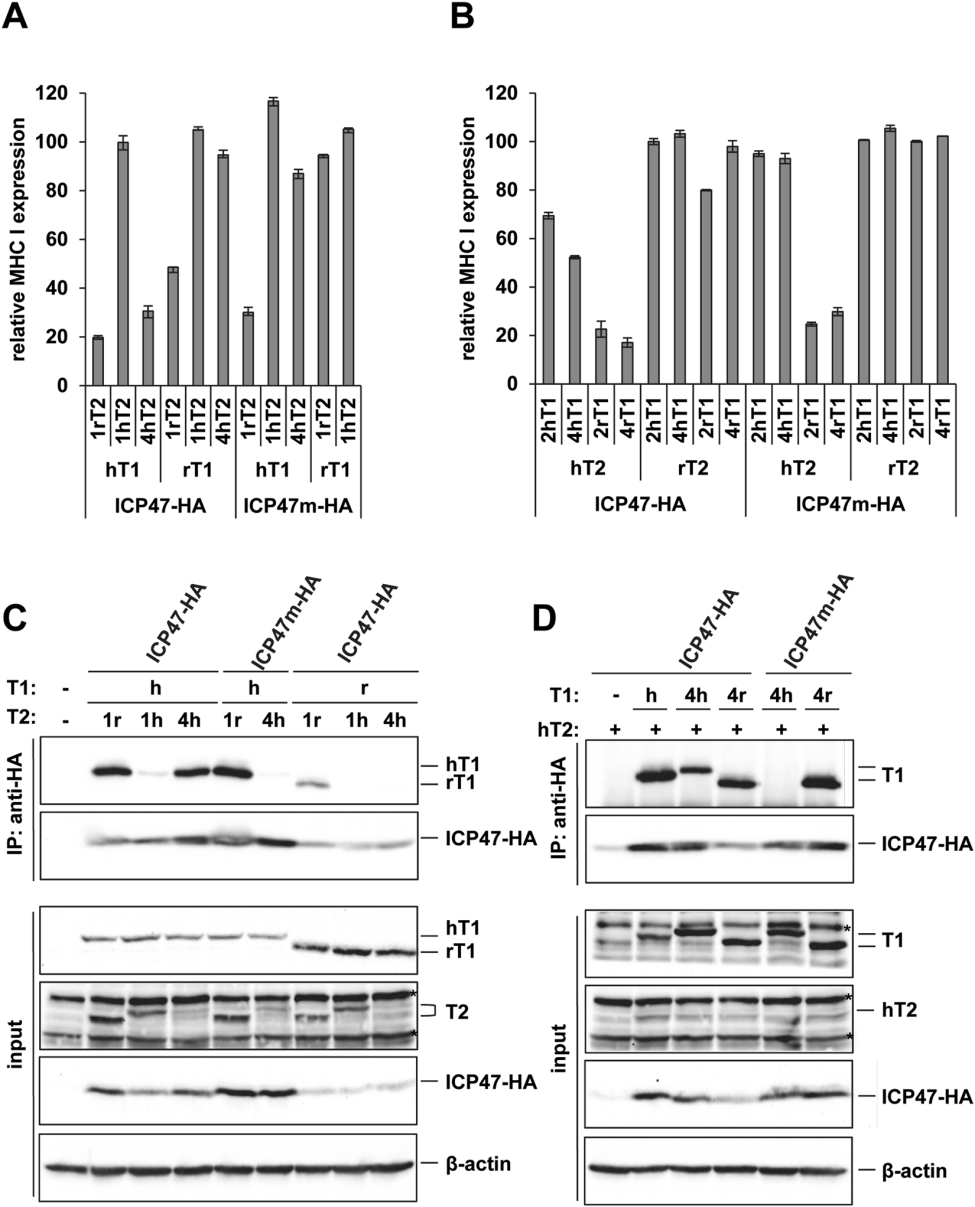
Divergent interaction sites of ICP47 on TAP1 and TAP2. Experiments were performed as in Fig. 4 with the indicated TAP chimeras (the FACS experiments shown in Figs 4 and 6 were performed in parallel). The figures (**A**,**C**) ﻿depict TAP2 chimeras (antibodies used for detection in B: TAP1, 148.3; ICP47-HA, anti-HA; TAP2 chimeras, anti-FLAG) and (**B**,**D**) TAP1 chimeras (antibodies used for detection in D: TAP1, anti-Myc; ICP47-HA, anti-HA; hTAP2, anti-FLAG). Original blots are shown in Fig. S10. Replicates of the co-IPs are shown in the Fig. S11.

The TAP1 chimeras were tagged with a Myc-tag at the C-terminus and the TAP2 chimeras with a FLAG-tag. The chimeras were stably expressed in CMT64.5 cells with either a human or rat counterpart. The expression levels of MHC class I on these cells varied between the different combinations of chimeric subunits (Fig. S9), however, the strength by which ICP47 downregulated MHC class I cell surface expression did not correlate with this (Fig. 6A and B).

Analysis of the TAP2 chimeras revealed more than one interaction site between ICP47 and TAP2. The chimera 1rT2 was similarly inhibited by ICP47-HA as WT hT2 in combination with either human or rat TAP1 (see Fig. 4A), indicating that the 1rT2 chimera contains all interactions sites found in the WT hT2 (Fig. 6A). Also the chimera 4hT2 was efficiently blocked in combination with hT1 by ICP47-HA, but inhibition was lost for ICP47m-HA, and for ICP47-HA when the chimera was co-expressed with rT1, suggesting that important interaction sites in both the regions of TMs 2–4 and 5–6 must exist. Inhibition by ICP47m-HA was only observed for hT1/1rT2 (Fig. 6A), which was similar to inhibition of hT1/hT2 (Fig. 4A). This finding was mirrored in the binding studies, with strong co-IP of the hT1/1rT2 combination by both WT ICP47 and the mutant (Fig. 6C). In accordance with inhibition of MHC class I expression, binding of WT ICP47-HA to the combinations rT1/1rT2 and hT1/4hT2 was also observed.

The use of TAP1 chimeras revealed a more narrow interaction with ICP47 than observed for TAP2. The chimeric TAP1 molecule 4rT1 mimicked hT1 in all aspects (Fig. 6B), also in combination with ICP47m-HA. We therefore concluded that the most important interaction sites for ICP47 on TAP1 are found in the region of TMs 5–6. Chimeras containing rat TMs 5–6 (2hT1, 4hT1) showed only intermediate inhibition by ICP47-HA when combined with hT2 (similar to rT1/hT2); ICP47m-HA, however, did not at all inhibit these chimeras. These results were confirmed by co-IP analysis of the chimeras 4rT1 and 4hT1 (Fig. 6D). Hence, whereas both N- and C-terminal portions of the TAP2 TMD was recognized by ICP47, interaction with TAP1 was only observed at the C-terminal TMD. This implies divergent recognition of human TAP1 and TAP2 by ICP47.

## Discussion

ICP47 encoded by HSV-1 was the first TAP inhibitor identified and its inhibitory mechanism has been extensively investigated. Previous studies indicated that the active domain of ICP47 is located within the first 35 N-terminal residues^29, 30^. Subsequent structural data revealed that this region adopts a helix-turn-helix conformation that inserts in to the pore formed by the TMDs of TAP1/TAP2^31, 34^ at a location that overlaps with the putative peptide-binding site suggested by mutagenesis and crosslinking experiments^36–39^. Thus it is now well established that ICP47 physically blocks binding of peptides to TAP. However, Galocha *et al*. have speculated that an additional stabilizing function could be conferred by the highly conserved sequence (34–52) of ICP47, which lies outside of the defined active domain. These authors commented that such an effect would not have been possible to measure using their particular experimental setting^29^. Here we confirm the existence of residues downstream of aa 35 that have a functional impact on TAP inhibition. Firstly, we used fusion proteins to analyze ICP47 truncation mutants, which strongly suggested that the residues 1–35 (ICP47-N-TG) do not possess the full inhibitory potential of ICP47. However, since fusion constructs of truncated ICP47 might cause inadvertent effects, we also mutated the conserved residues _50_PLL_52_ of ICP47 full-length HA-tagged protein to alanine residues that are supposed to be functionally neutral (ICP47m-HA). The fact that the mutant ICP47m-HA and the fusion protein ICP47-N-TG only poorly blocked MHC class I cell surface expression, but to a similar extent, indeed supported the hypothesis that the function lacking in the ICP47-N-TG fusion protein is conferred by residues 50–52 of ICP47.

US6 is a HCMV-encoded type I glycoprotein that inhibits TAP from the luminal side of the ER membrane. Previously, we analyzed binding sites of US6 on TAP, which suggested that US6 does not recognize the luminal loops of TAP, but more likely dips into the translocation pore to interact with residues of the TAP1 and TAP2 TMDs^3^. In addition, US6 binds to TAP in a peptide-bound state^21^, implying recognition of a conformation distinct to that stabilized by ICP47. We therefore hypothesized that US6 would not be able to bind TAP complexes, if ICP47 was allowed to first freeze their conformation. Indeed this was the case, which clearly demonstrates that ICP47 and US6 bind to TAP during different steps of the peptide translocation cycle. We also performed the reciprocal experiment and likewise observed that prior expression of US6 strongly blocked ICP47 binding to TAP (Fig. S12). Therefore, ICP47 and US6 are mutually exclusive and block the progression of the transport cycle by freezing TAP in distinct conformations. In contrast, neither the fusion protein ICP47-N-TG nor ICP47m-HA were able to block US6 binding to TAP, even though both proteins bound to TAP. This suggests that without the highly conserved central region ICP47 can still bind TAP and inhibit TAP to a limited extent, but is unable to confer arrest of the TAP cycle. Recently, another study reported a thermostabilizing effect on TAP exerted by the residues 35–55 of ICP47^40^. These residues were suggested to be important for a conformational arrest of TAP and therefore strongly support our data.

Thus, we propose a revised model of TAP inhibition by ICP47, whereby two distinct mechanisms that are encoded by different regions of ICP47, contribute to full TAP inhibition. Here, the N-terminus of ICP47 (residues 2–35) blocks the binding of peptides to TAP, whereas the central region (residues 50–52) contributes to a conformational freezing of the TAP translocation cycle. This model is entirely consistent with the near-atomic resolution (4 Å) structure of TAP bound to ICP47^34^ that has been reported while this manuscript was under review. Importantly, the improved resolution of this structure has now allowed the amino acid register of ICP47 to be determined. Notably, ICP47 residues 50–52 were well resolved and described to adopt a hairpin-like conformation, the apex of which sits alongside a region that forms the transmission interface for conformational communication between the NBDs and the TMDs, and makes extensive interactions with the TAP2 CH1 coupling helix (Fig. S13)^12^. Thus, the intimate apposition of ICP47 residues 50–52 to a region known to be involved in conformational change of TAP provides a likely mechanism for understanding how ICP47 freezes peptide translocation by TAP. In support of this proposition, crosslinking of CH1 to the X-loop has previously been reported to arrest the TAP cycle and block peptide transport^12^.

**Figure 7:**
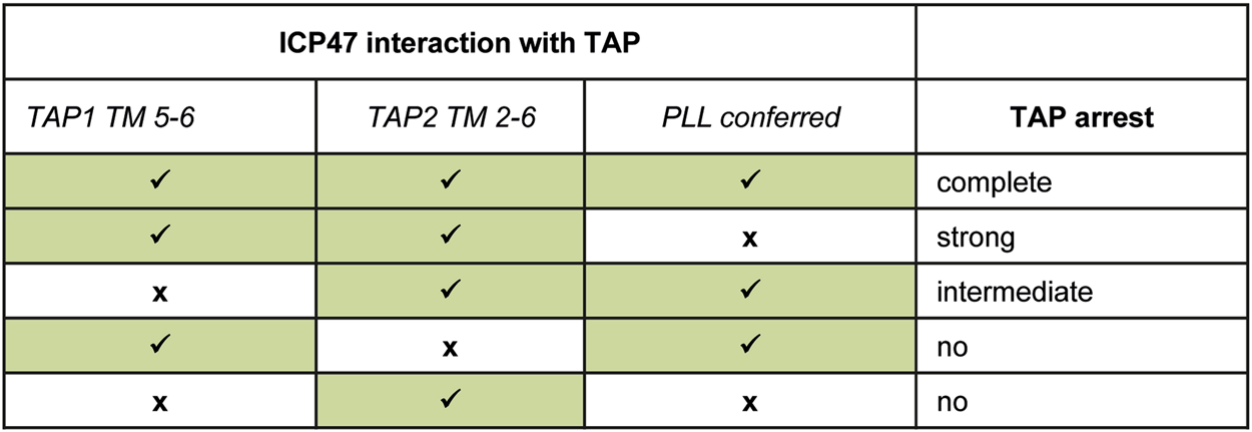
Functional hierarchy of ICP47 interaction sites on TAP. Colored cells indicate that interaction occurs with TAP1 TM5-6, TAP2 TM2-6 or via the _50_PLL_52_ motif. Arrest of the TAP complex requires interaction with TAP1, TAP2 and the _50_PLL_52_ mediated stabilization. Lack of the _50_PLL_52_ motif still allows inhibition (strong) but the TAP cycle cannot be completely blocked and undergoes a delayed progression. If interaction with TAP1 is missing, but interaction sites on TAP2 are present in combination with the _50_PLL_52_ sequence, TAP is blocked with an intermediate efficiency. Lack of TAP2 interaction, or of TAP1 and _50_PLL_52_ simultaneously, leads to loss of inhibition.

To understand further how ICP47 residues 50–52 might influence the interaction between ICP47 and TAP, we took advantage of the species-specificity of ICP47 and analyzed inhibition and binding to human and rat TAP subunits. Combining human or rat TAP subunits only, we were able to confirm the species-specific nature of ICP47. Substitution of rat TAP1 with human TAP1 did not alter this outcome, whereas replacement of rat TAP2 did, resulting however, in a partial rescue of the inhibitory effect. This indicates that interactions with TAP2 are essential for ICP47 function, but that interaction sites on TAP1 also make crucial contributions for full inhibition. Interestingly, the Pro_50_Leu_51_Leu_52_ to AlaAlaAla mutation of ICP47 led to a loss of the observed partial TAP inhibition via hTAP2 when combined with the rTAP1 subunit. Inhibition by the ICP47 mutant was observed exclusively when both TAP1 and TAP2 were of human origin. From this we conclude that ICP47 interacts with the TMDs of both TAP1 and TAP2. These interactions sites do not contribute equally to the inhibitory efficiency of ICP47, but seem to have hierarchical influences (Fig. 7), as discussed below.

In the structural model by Oldham *et al*. the N-terminus of ICP47 forms a hairpin-like structure within the TAP translocation pore, thereby contacting TMs of both TAP1 and TAP2^31, 34^. Our study of chimeric TAP subunits confirmed the interactions with TAP1 and TAP2. Using chimeric human/rat TAP2 subunits we observed two regions important for ICP47 binding and full TAP inhibition (TM2-4 and TM5-6). These findings are in good agreement with the structural ICP47-TAP model. Interaction with the region of the TMs 2–4 of TAP2 is sufficient to mediate inhibition when concurrent binding to TAP1 and interaction via the _50_PLL_52_ site occurs. However, if one of the latter two sites is missing, inhibition is lost. Additional interaction with the region of TMs 5–6 of TAP2, however, leads to an inhibitory effect even in the absence of either TAP1 or the _50_PLL_52_ sequence.

Analysis of ICP47 binding and inhibition of TAP1 chimeras suggested a more selective interaction with TAP1. In particular, using chimeric human/rat TAP1 subunits, only the region of TMs 5–6 (4rT1) was found to be important for ICP47 inhibition. This finding is consistent with the recently published structural model^34^, which suggests that Gln456 from the TAP1 TM6 could potentially form interactions with Asn14 and Arg16 within the ICP47 interconnecting loop, although the resolution was not sufficient to accurately determine the side chain conformations in this region. Nevertheless, it is interesting to note that residue 456 in rat TAP1 is an arginine, and the presence of a basic residue in this position could potentially lead to charge repulsion with Arg16 of ICP47, thereby explaining why ICP47 does not bind to rat TAP1. Interestingly, in our previous study focused on US6 the 4rT1 chimera was efficiently blocked by US6^3^. This raises the intriguing question as to whether ICP47 and US6 could target TAP1 at the same position with US6 possibly reaching downwards into the open pore in the TAP outward-facing conformation, and ICP47 contacting the same position from the cytosolic side but in the inward-facing conformation.

In summary, our study for the first time demonstrates a complex two-tiered mechanism of TAP inhibition by ICP47, involving not only the previously described blocking of peptide binding to TAP^27, 28^ but also an additional ICP47-TAP interaction that results in a freezing of TAP conformational change, likely *via* a stabilization of the TAP2 coupling helix. Understanding of the mechanisms by which ICP47 blocks conformational changes of TAP will give important insights into how ABC transporters transmit structural changes in the TMDs to the NBDs. Precise identification of the site targeted by the residues 50–52 of ICP47 on the TAP peptide transporter will possibly open new avenues for the development of general inhibitors of ABC transporters.

## Methods

### Cells and antibodies

HeLa (ATCC CCL-2), CMT64.5^41^ and HEK293T (ATCC CRL-1573) cells were grown in DMEM supplemented with 10% FCS, penicillin and streptomycin. When indicated, cells were transfected using Superfect (Qiagen) following the manufacturer’s protocol. In this study the following antibodies were applied: rabbit anti-TagGFP (Evrogen); the mAb 148.3 for TAP1 detection (kindly supplied by E. Wiertz, Utrecht)^42^; rabbit anti-calnexin (Stressgen) and mouse anti-β-actin (Sigma-Aldrich); the mAb W6/32 for the detection of fully conformed, peptide-loaded HLA-A,B,C molecules^43^; APC-coupled anti-mouse antibodies (BD Pharmingen); anti-mouse anti-HA antibodies for flow cytometry and Western blot analysis (Sigma-Aldrich); rabbit anti-HA for immunoprecipitation (Sigma-Aldrich); anti-c-Myc and anti-FLAG clone M2 (Sigma-Aldrich); the mAb 20-8-4S specific for H-2K^b^ and H-2D^b^ ^44^. The polyclonal anti-US6 serum was described before^3^.

### Lentiviral transduction

Production of lentiviruses was performed as described previously^45^. The transduced cells were cultivated in normal medium for 1–2 days before treatment with puromycin (5 g/ml, Sigma-Aldrich) or with geneticin (1 mg/ml, Sigma-Aldrich).

### Cloning

The ICP47-TagGFP fusion constructs were cloned into pcDNA3.1. First TagGFP was cloned into BamHI and XhoI. ICP47 C-terminal truncation mutants were amplified without a stop codon and the sequence at the 3′-end encoding the linker SGA_4_GGSGS and were cloned into NheI and BamHI sites after amplification using the forward primer 5′-gctgctagcatgtcgtgggccctggaa-3′ for all constructs and the reverse primer 5′-cgaggatccgctgcctcctgcagcggccgctccggacgcataatccggcacatcatacgg-3′ for ICP47-WT-TG, 5′-cgaggatccgctgcctcctgcagcggccgctccggactcacgcccccttttattgatctcatcg-3′ for ICP47-N-TG, and 5′- cgaggatccgctgcctcctgcagcggccgctccggagggagagcgcagcagagg-3′ for ICP47-eN-TG. To allow for lentiviral transduction, the ICP47-TagGFP constructs were subcloned into the NheI site of puc2CL6Ipwo vector after excising the fusion constructs with NheI and XbaI from pcDNA3.1. Cloning of ICP47-HA into pcDNA3.1 was described before^46^. ICP47m-HA was produced by site directed mutagenesis using the Q5 Site-Directed Mutagenesis Kit (NEB). US6-HA was cloned into NheI and BamHI of pIRES-EGFP using the primers 5′-cgtgctagcatggatctcttgattcgtctcg-3′ and 5′-gcaggatccttacgcgtaatctggaacatcgtatgggtaagcggagccacaacgtcgaatc-3′.

For construction of HA-tagged MHC class I, the tapasin signal peptide sequence was amplified with a HA-tag sequence using the primers 5′-cgtctcgagatgaagtccctgtctctgctcc-3′ and 5′-cctactgcagctgcgtaatctggaacatcgtatgggtacaccgcgggtcctgctga-3′and cloned into XhoI and PstI sites of pIRES-EGFP (Tpn-SP-pIRES-EGFP). HLA-B*44:02 was amplified with the primers 5′-cgtatgcattaggaggctcccactccatgagg-3′ and 5′-cgaggatccttcaagctgtgagagacacatc-3′ and HLA-B*15:03 with 5′-cgtatgcattaggaggctcccactccatgagg-3′ and 5′-cgaggatcctcaagctgtgagagacacatcag-3′ and cloned into PstI (PCR products were digested with NsiI) and BamHI sites of Tpn-SP-pIRES-EGFP. Sequenced inserts were subsequently subcloned into the puc2CL6Ipwo lentiviral vector using the restriction sites NheI and BamHI. Lentiviral vectors were also used for transient expression of MHC class I molecules.

Cloning of human and rat TAP1 and TAP2 subunits and chimeras thereof was described before^3^. The names of the constructs used in the previous study and the epitope tag added in this one are given in Supplemental Table I. For cloning of rT1 into the XhoI and NheI sites of the puc2CL6Ipwo vector the primer pair 5′-gctgtcgacgccgccaccatggctgcgcacgcctgg-3′ and 5′-cgagctagctcattctggagcatctgcaggagc-3′ was used. For cloning of the other TAP1 subunits the forward primer 5′-gctgtcgacgccgccaccatggctagctctaggtgtcccg-3′ was used for sequences with a human 5′-end and the primer 5′-gctgtcgacgccgccaccatggctgcgcacgcctgg-3′ for sequences with a rat 5′-end. TAP1 subunits with a human 3′-end were amplified with the reverse primer 5′-cgaagatcttcacagatcctcttctgagatgagtttttgttcttctggagcatctgcaggagcc-3′ and those with a rat 3′-end with 5′-cgaagatcttcacagatcctcttctgagatgagtttttgttcgtctgaaggagccgcaagagc-3′. TAP2 subunits with a human 5′-end were amplified with the forward primer 5′-gctctcgaggccgccaccatgcggctccctgacctg-3′ and those with a rat 5′-end with 5′-gctctcgaggccgccaccatggcgctgtcctacccg-3′. The reverse primers 5′-cgagctagcctacttgtcgtcatcgtctttgtagtcgagctgggcaagcttctgc-3′ and 5′-gagctagcctacttgtcgtcatcgtctttgtagtccgcctccagccgctgctg-3′ were used for human and rat 3′-ends respectively. TAP1 subunits were cloned into the XhoI and BamHI sites of puc2CL6Ipwo. TAP2 subunits were cloned into the XhoI and NheI of puc2CL6Inwo.

### Flow cytometry analysis

Cells were trypsinized and stained with antibodies diluted in 3% FCS/PBS. Cells were washed in 3% FCS/PBS, supplemented with DAPI and measured by FACS Canto II (Becton Dickinson). Acquired data was analyzed by FlowJo (v10.1, Tree Star Inc.).

### Immunoprecipitation and Western blot analysis

Cells were detached by scraping and washed with PBS. Ca. 3 × 10^6^ cells were lysed in 0.5 ml of digitonin lysis buffer (140 mM NaCl, 20 mM Tris [pH 7.6], 5 mM MgCl_2_, and 1% digitonin (Calbiochem)) for 30 min and cleared from membrane debris at 13,000 rpm for 30 min at 4 °C. Lysates were incubated with antibodies for 3–4 h at 4 °C in an overhead tumbler before immune complexes were retrieved by protein A-Sepharose (GE Healthcare). Sepharose pellets were washed four times with increasing NaCl concentrations (0.15 to 0.5 M in lysis buffer containing 0.2% detergent). Retrieved proteins were separated by SDS-PAGE and blotted onto a nitrocellulose membrane. Proteins were detected after treatment with specific antibodies followed by peroxidase-conjugated secondary antibodies and the SignalFire ECL Reagent (Cell Signaling Technologies). The Biorad Image Lab software was used for quantification of band intensities measured with a Biorad Chemidoc XRS system. Blots were rotated and cropped for convenient presentation of data using Adobe Photoshop CS5. Examples of the procedure used for presentation of blots are shown in Figs 1C, S3, 4B and S7.

### Peptide translocation

Peptide translocation was performed as previously described^47^. 2 × 10^5^ HeLa cells were semi-permeabilized using streptolysin O (0.15 µg/ml; Abcam). For permeabilization with SLO HeLa cells were incubated at 4 °C for 15 min and then washed in PBS to remove residual SLO. Transport was carried out in the presence of 10 mM ATP/ADP, 5 nM NST-Cy5 in PBS buffer supplemented with 10 mM of MgCl_2_ for 15 min at 37 °C in 50 μl. The reaction was stopped by addition of 150 µl PBS supplemented with EDTA (20 mM). Samples were acquired with the LSR II (BD) and analyzed using FlowJo 7.6.5 software reporting the mean fluorescence intensity (MFI). For HeLa cells expressing the ICP47 fusion proteins, MFI was analyzed from GFP positive cells only. The NST-Cy5 (RRYQNSTC(Cy5)L, cysteine labeled with Cy5) peptide was synthesized by the platform “Peptide Synthesis” of the Helmholtz Centre for Infection.

## Electronic supplementary material

Supplementary Information
