# Supplementary material for: A highly conserved sequence of the viral TAP inhibitor ICP47 is required for freezing of the peptide transport cycle
Source: Sci Rep. 2017 Jun 7;7:2933. doi: 10.1038/s41598-017-02994-5 (PMC5462769; doi:10.1038/s41598-017-02994-5)
Supplement: Supplementary file 1 — Supplementary Information [file 41598_2017_2994_MOESM1_ESM.docx]

**Supplementary Information:**

**A highly conserved sequence of the viral TAP inhibitor ICP47 is required for freezing of the peptide transport cycle**

Tony Matschulla, Richard Berry, Carolin Gerke, Marius Döring, Julia Busch, Jennifer Paijo, Ulrich Kalinke, Frank Momburg, Hartmut Hengel, Anne Halenius


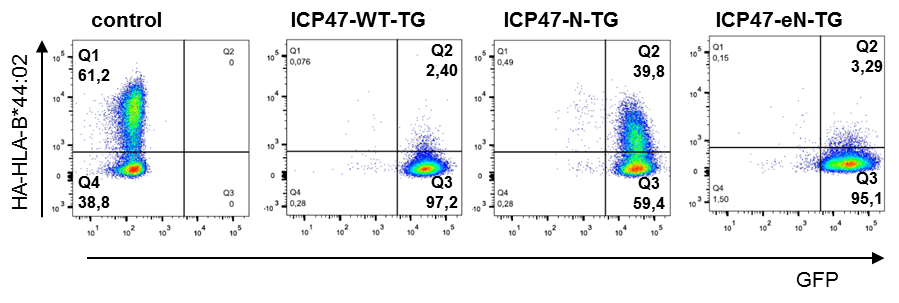


**Supplementary Figure 1.** HeLa cells stably expressing fusion proteins as indicated were transiently transfected with a HA-HLA-B*44:02 expression plasmid. At 20 h post-transfection cell surface expression was analyzed by flow cytometry using anti-HA antibodies.

**
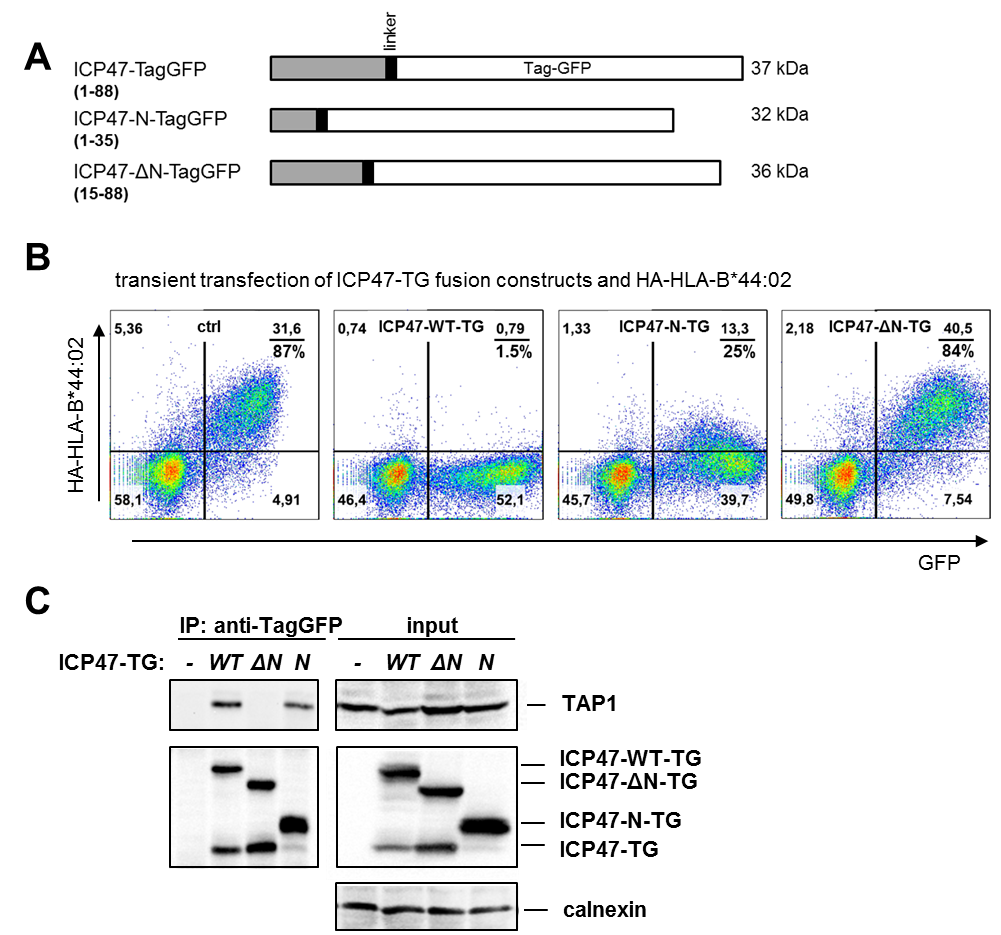
**

**Supplementary Figure 2. The ICP47-ΔN-TG mutant does not affect MHC class I cell surface expression.** (**A**) Schematic depiction of ICP47-TagGFP fusion proteins with a C-terminal linker and TagGFP, and apparent protein sizes. The ICP47-ΔN-TagGFP fusion construct was cloned into pcDNA3.1 as described for the other TG fusion constructs (see Methods part). For amplification of ΔN-ICP47 the forward primer 5’- gctgctagcatgcgggttgggcccagg -3’ and the reverse primer 5’- cgaggatccgctgcctcctgcagcggccgctccggaacgggttaccggattacg-3’ was used. (**B**) HeLa cells were transiently transfected with fusion proteins shown in A and HA-tagged HLA-B*44:02. At 20 h post-transfection cell surface expression was analyzed by flow cytometry using anti-HA antibodies. The percentages in the upper right corner, give the percentage of cells in this square from the total pool of GFP-positive cells. (**C**) HeLa cells stably expressing fusion proteins shown in A were lysed in digitonin lysis buffer and an anti-TagGFP IP was performed. Aliquots of the lysates (input) and recovered proteins (IP) were separated by SDS-PAGE and detected by Western blotting (TAP1, 148.3; ICP47-TG fusion constructs, anti-TagGFP). One representative experiment out of two independent experiments is shown.

**
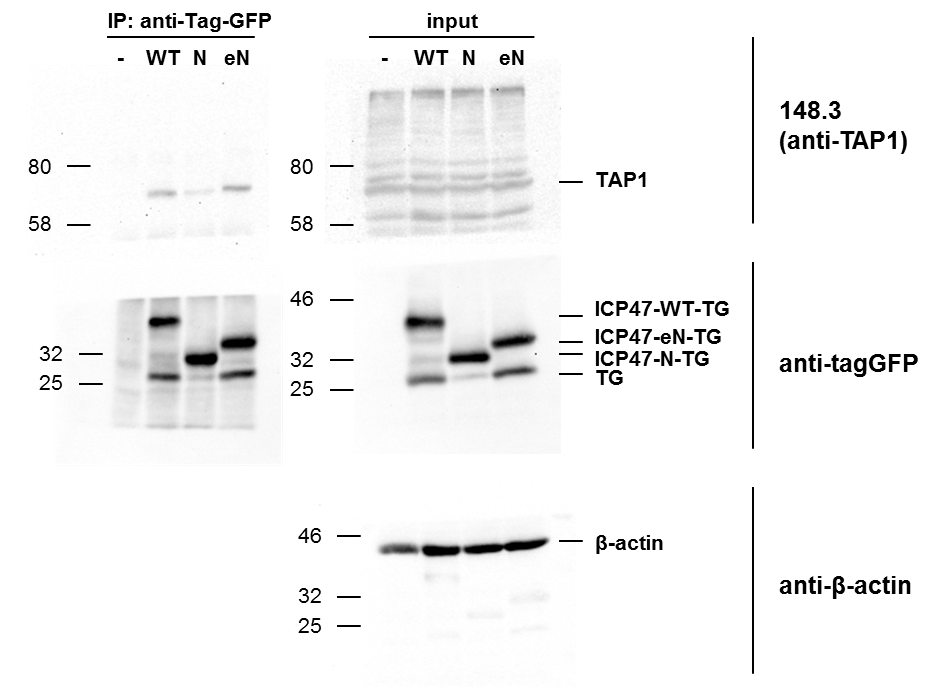
**

**Supplementary Figure 3.** Cropped blots from Fig. 1C are shown in original format. Prior to detection the region of the blot containing the HC of the antibody used for the IP was cut out. The upper part of the blot (58-80 kDa) was used for detection of TAP1, the lower part (25-46 kDa) for detection of ICP47-TG constructs. After anti-tagGFP detection the lower part of the blot was treated with an antibody stripping solution and subsequently β-actin was detected.

**
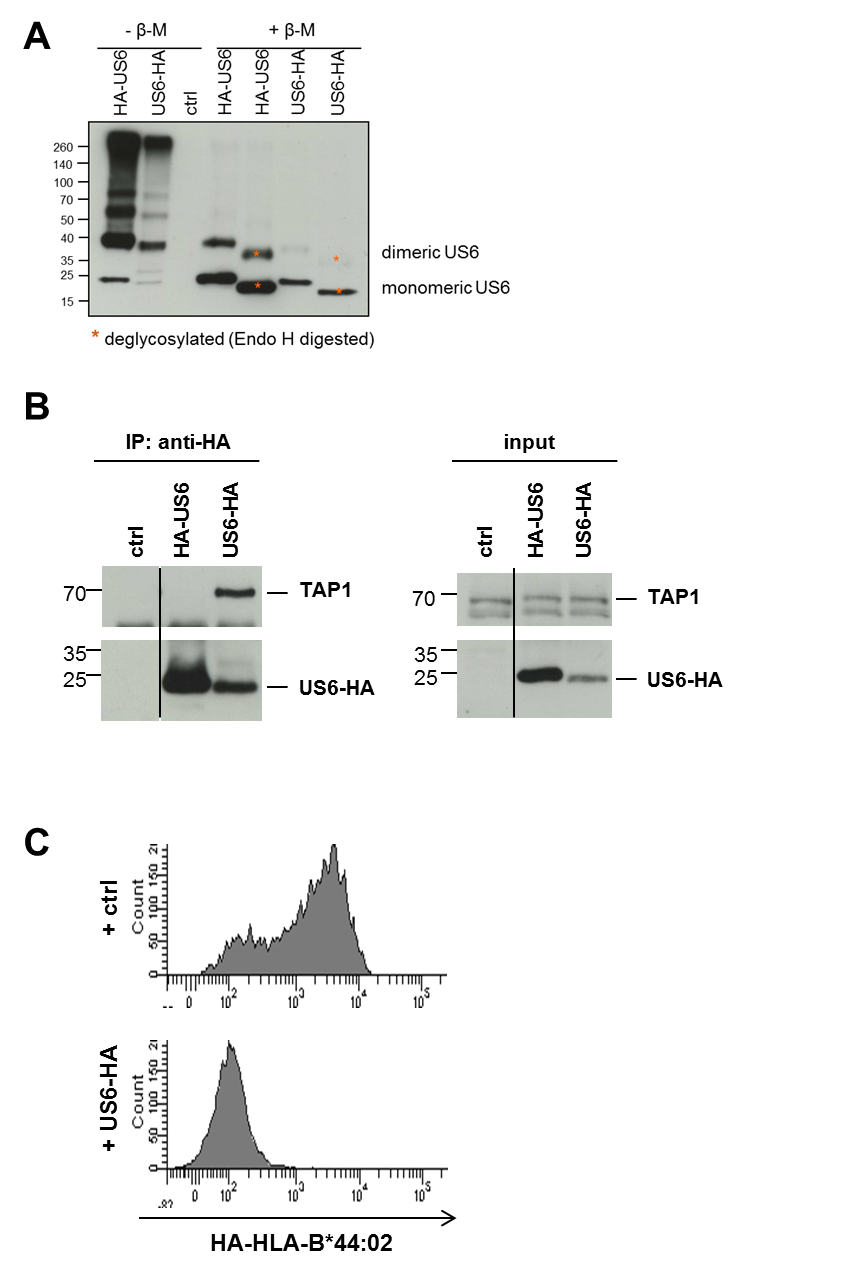
**

**Supplementary Figure 4. US6-HA binds to TAP and block MHC class I expression.** (**A**) N- and C-terminally HA-tagged US6 constrcuts were transiently transfected into HeLa cells (for cloning of N-terminally HA-tagged US6 the primers 5’-cgtctgcaggtgagcggtcttcgcgtg-3’ and 5’-gcaggatcctcaggagccacaacgtcgaatc-3’ were used to amplify US6 without a sequence for the signal peptide and the PCR product was cloned into the restriction sites PstI and BamHI of the vector Tpn-SP-pIRES-EGFP (see Methods part).). At 20 h post-transfection cell lysates were prepared and these were left untreated or were treated with Endoglycosidase H (orange asterisk). Proteins were separated by SDS-PAGE after boiling of lysates with or without β-mercaptoethanol (β-M) and detected by Western blotting using an anti-HA antibody. (**B**) Performed as in A: At 20 h post-transfection HeLa cells were lysed in digitonin lysis buffer and an anti-HA IP was performed. Aliquots of the lysates (input) and recovered proteins (IP) were separated by SDS-PAGE and detected by Western blotting (TAP1, 148.3; HA-tagged US6 proteins, anti-HA). HeLa cells were transiently transfected with fusion proteins shown in A and HA-tagged HLA-B*44:02. (**C**) A control or US6-HA construct was transiently transfected into HeLa cells together with an HA-tagged HLA-A*B44:02. At 20 h post-transfection cell surface expression of HA-HLA-B*44:02 was analyzed by FACS using an anti-HA antibody.

**
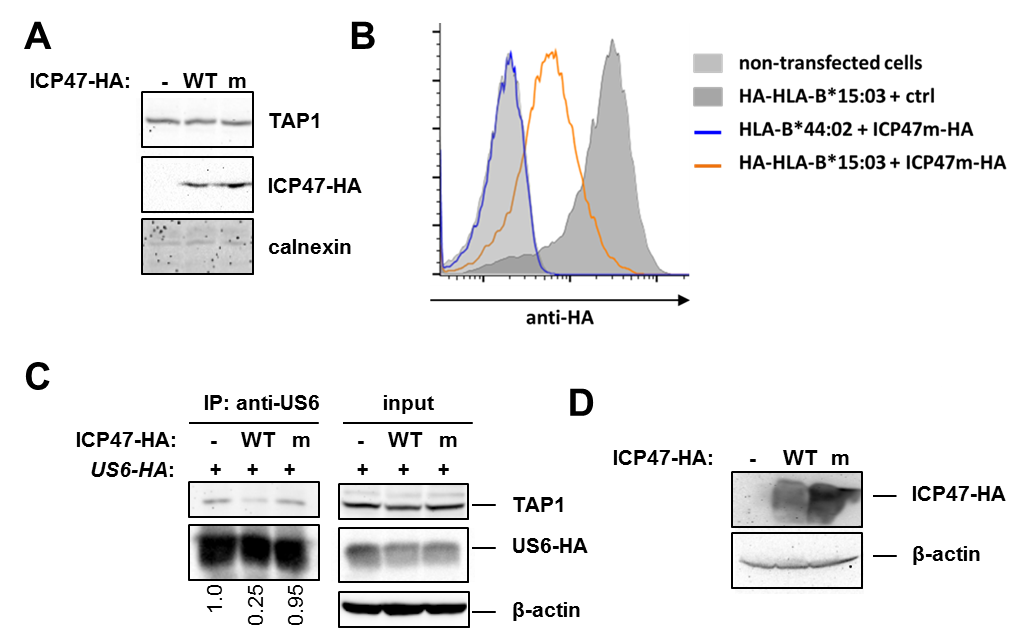
**

**Supplementary Figure 5. Reduced blocking of US6-HA binding to TAP by ICP47m-HA**. (**A**) ICP47-HA and ICP47m-HA and were transiently transfected into HeLa cell. Expression wsa analyzed by Western blotting using anti-HA antibodies. (**B**) HeLa cells were transiently transfected as indicated. At 20 h post-transfection staining of the cell surface using an anti-HA antibody was analyzed by FACS. (**C**) Control HeLa cells (ctrl) or HeLa cells stably expressing WT or mutant (m) ICP47-HA were transiently transfected with US6-HA. At 20 h post-transfection cells were lysed in digitonin lysis buffer and an IP was performed using anti-US6 antibodies. Aliquots of the lysates (input) and recovered proteins (IP) were separated by SDS-PAGE and detected in Western blot (TAP1, 148.3; US6-HA, anti-HA). The relative intensity of the TAP1 band compared to the US6-HA band in the IP samples is given below the upper panel. The value of the ctrl sample was set to 1. (**D**) Cell lysates from stable cell lines used in C were analyzed by Western blotting as indicated (ICP47-HA, anti-HA).

**
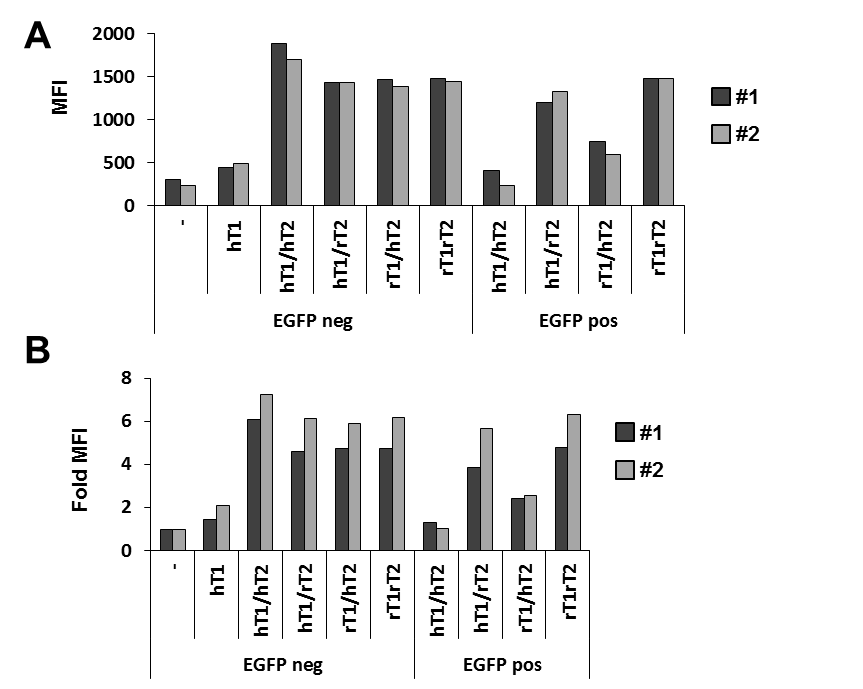
**

**Supplementary Figure 6.** (**A**) The diagram depicts MFI values of the EGFP negative (ICP47-HA negative) and EGFP positive (ICP47-HA positive) cells from the flow cytometry analysis shown in Figure 4A. Two independent experiments are shown (#1 and #2). (**B**) The diagram depicts relative MFI values from the FACS analysis in A. The MFI value of each sample is shown relative to the MFI of CMT64.5 (-) cells in that analysis. The value of CMT64.5 (-) cells were set to 1. Only the pool of ICP47-HA-transduced cells is shown.

**
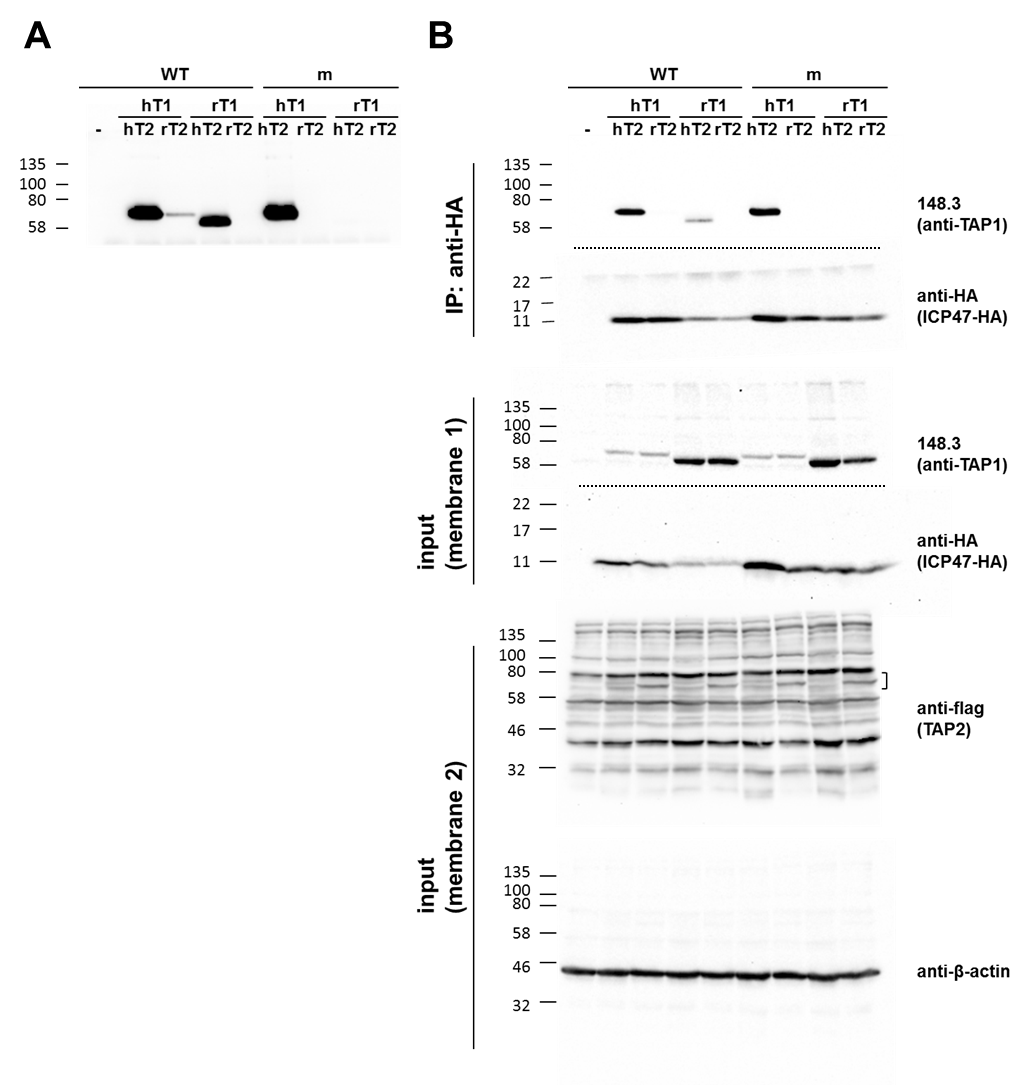
**

**Supplementary Figure 7.** (**A**) Long exposure of the TAP1 detection after anti-HA IP shown in Fig 4B. (**B**) Cropped blots from Fig. 4B are shown in original format. Curved brackets mark specific TAP2 bands. The dotted line indicates that the membranes were cut in parts before detection with specific antibodies (upper part 58-135 kDa, lower part 11-22 kDa, the middle part was not used for detection of proteins). The membrane with the IP samples and membrane 1 were blotted with gels poured with a lower part containing 14% polyacrylamide (PAA) and an upper part containing 10% PAA. Membrane 2 was blotted with a 10% PAA gel. After anti-FLAG detection the blot was treated with an antibody stripping solution and subsequently β-actin was detected.

**
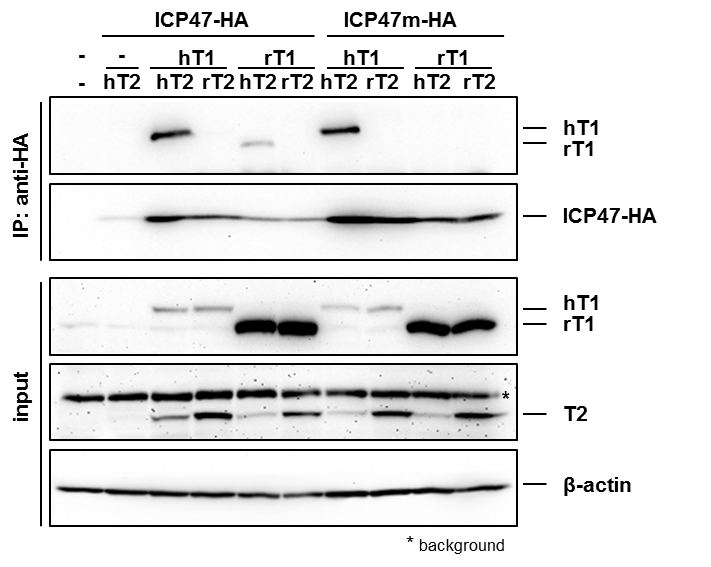
**

**Supplementary Figure 8.** Experimental replicate of co-IP shown in Figure 4B.

**
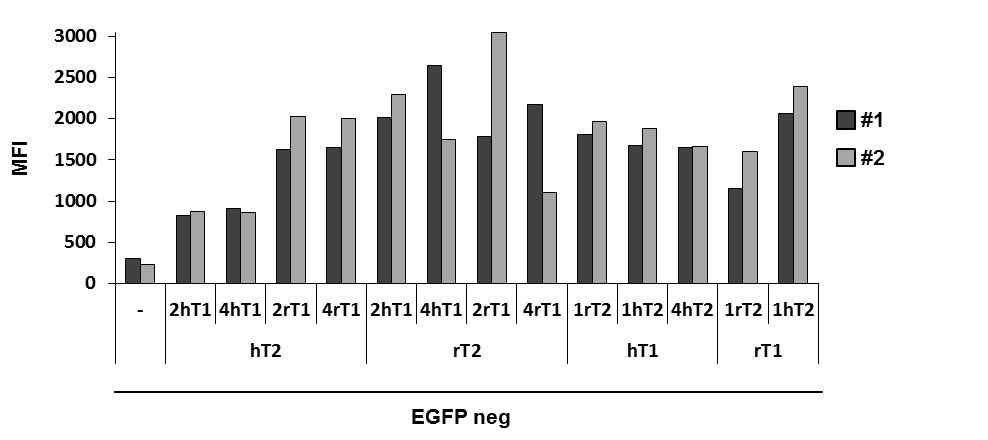
**

**Supplementary Figure 9.** The diagram depicts MFI values of the EGFP negative (ICP47-HA negative) cells from the flow cytometry analysis shown in Figure 6A and C. Two independent experiments are shown ((#1 and #2). Since the analysis shown in Fig. S5 and this analysis were performed in parallel the values of CMT65.4 (-) cells are identical.

**
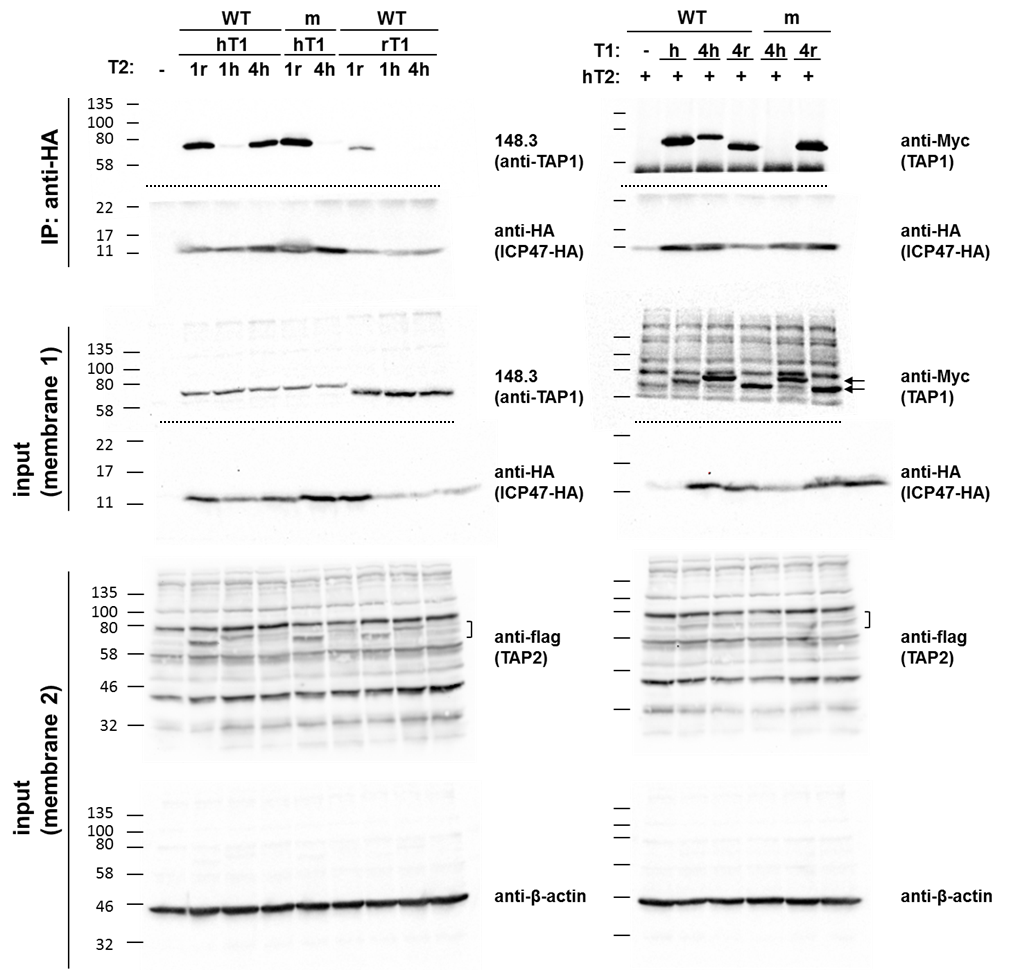
**

**Supplementary Figure 10.** Cropped blots from Figure 6B (left panel) and D (right panel) are shown in original format. Curved brackets mark specific TAP2 bands and arrows TAP1 chimeras. The dotted line indicates that the membranes were cut in two parts before detection with specific antibodies (upper part 58-135 kDa, lower part 11-22 kDa, the middle part was not used for detection of proteins). The membranes with the IP samples and membrane 1 were blotted with gels poured with a lower part containing 14% polyacrylamide (PAA) and an upper part containing 10% PAA. Membrane 2 was blotted with a 10% PAA gel. After anti-FLAG detection the blot was treated with an antibody stripping solution and subsequently β-actin was detected.

**
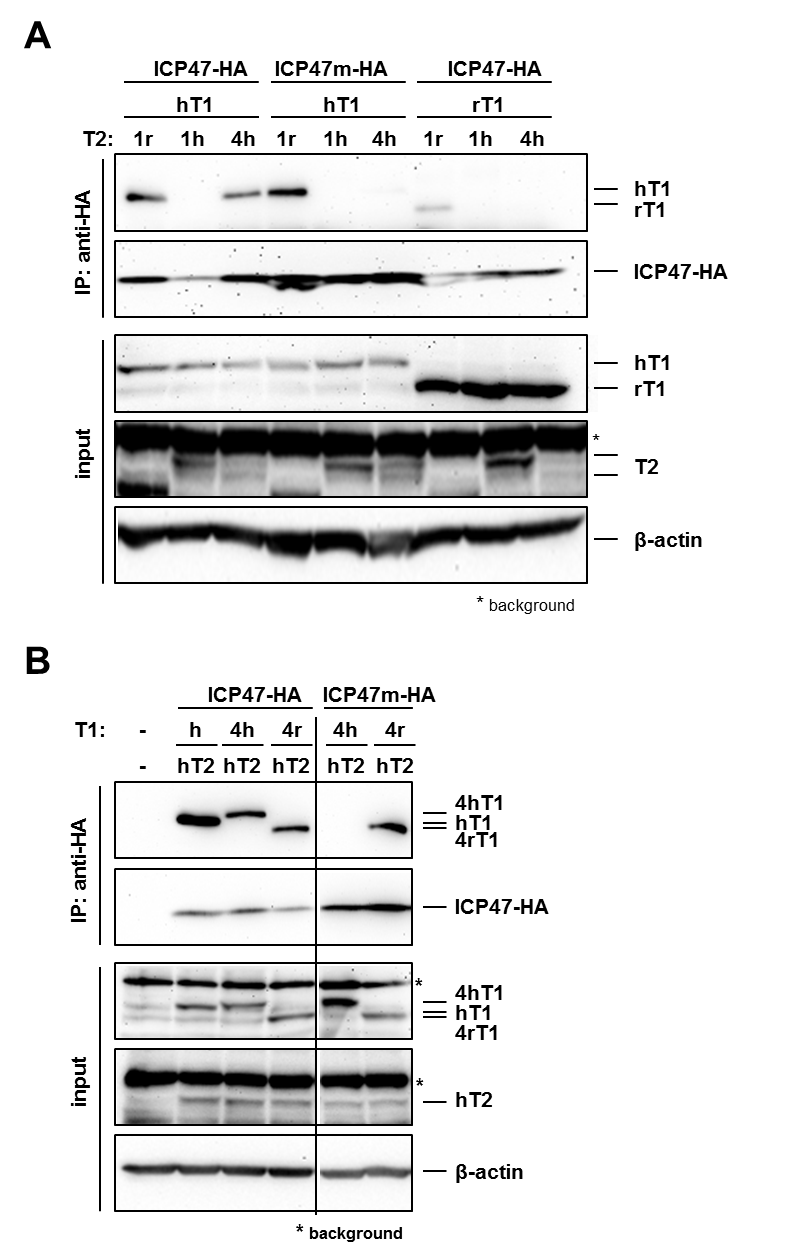
**

**Supplementary Figure 11.** (**A**) Experimental replicate of co-IP shown in Figure 6B. (**B**) Experimental replicate of co-IP shown in Figure 6D.


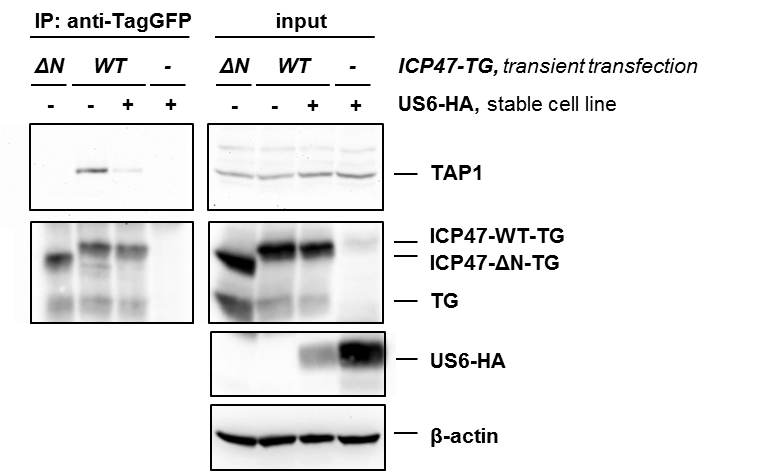


**Supplementary Figure 12. US6-HA blocks ICP47-WT-TG binding to TAP**. Control HeLa cells (-) or HeLa cells stably expressing US6-HA (+) were transiently transfected with ICP47-TG constructs as indicated. At 20 h post-transfection cells were lysed in digitonin lysis buffer and an IP was performed using anti-TagGFP antibodies. Aliquots of the lysates (input) and recovered proteins (IP) were separated by SDS-PAGE and detected in Western blot (TAP1, 148.3; ICP47-TG constructs, anti-TagGFP; US6-HA, anti-HA).


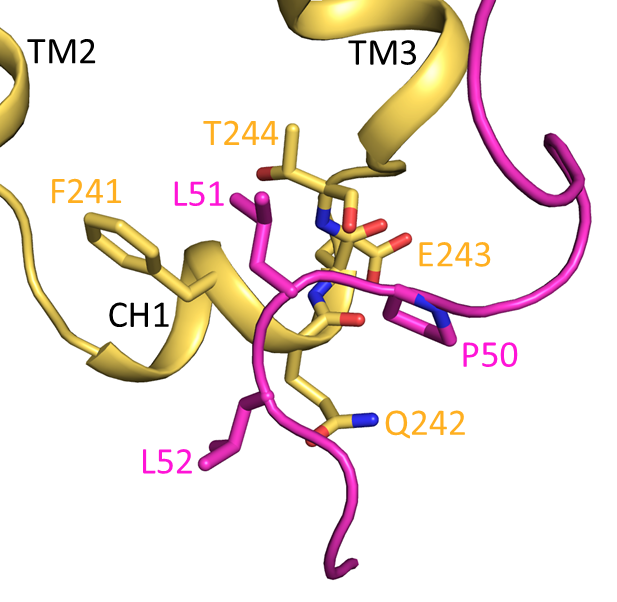


**Supplementary Figure 13. Interaction of ICP47 (magenta) residues 50-52 with TAP2 (gold).** The coordinates are derived from PDB ID: 5U1D.

**Supplementary Table I.**

Names of TAP1 and TAP2 subunits used in this study and described elsewhere[^1^](#_ENREF_1).

| This study | Halenius et al[^1^](#_ENREF_1) | Epitope tag |
| --- | --- | --- |
| hT1 | hT1 | Myc |
| rT1 | rxhT1 |  |
| 2rT1 | r6hT1 | Myc |
| 2hT1 | h6rT1 | Myc |
| 4rT1 | r8hT1 | Myc |
| 4hT1 | h8rT1 | Myc |
| hT2 | hT2 | FLAG |
| rT2 | rT2 | FLAG |
| 1rT2 | r4hT2 | FLAG |
| 1hT2 | h4rT2 | FLAG |
| 4hT2 | h7rT2 | FLAG |

1 Halenius, A. *et al.* Physical and functional interactions of the cytomegalovirus US6 glycoprotein with the transporter associated with antigen processing. *The Journal of biological chemistry* **281**, 5383-5390, doi:10.1074/jbc.M510223200 (2006).
